# Fertility Awareness-Based Methods for Women's Health and Family Planning

**DOI:** 10.3389/fmed.2022.858977

**Published:** 2022-05-24

**Authors:** Marguerite Duane, Joseph B. Stanford, Christina A. Porucznik, Pilar Vigil

**Affiliations:** ^1^Department of Family Medicine, Georgetown University, Washington, DC, United States; ^2^Fertility Appreciation Collaborative to Teach the Science (FACTS), Washington, DC, United States; ^3^Office of Cooperative Reproductive Health, Division of Public Health, Department of Family and Preventive Medicine, University of Utah, Salt Lake City, UT, United States; ^4^Reproductive Health Research Institute (RHRI), New York, NY, United States

**Keywords:** fertility awareness, women's health, family planning, infertility, menstrual cycle, fertility apps, natural family planning, reproductive health

## Abstract

**Background:**

Fertility awareness-based methods (FABMs) educate about reproductive health and enable tracking and interpretation of physical signs, such as cervical fluid secretions and basal body temperature, which reflect the hormonal changes women experience on a cyclical basis during the years of ovarian activity. Some methods measure relevant hormone levels directly. Most FABMs allow women to identify ovulation and track this “vital sign” of the menstrual or female reproductive cycle, through daily observations recorded on cycle charts (paper or electronic).

**Applications:**

Physicians can use the information from FABM charts to guide the diagnosis and management of medical conditions and to support or restore healthy function of the reproductive and endocrine systems, using a restorative reproductive medical (RRM) approach. FABMs can also be used by couples to achieve or avoid pregnancy and may be most effective when taught by a trained instructor.

**Challenges:**

Information about individual FABMs is rarely provided in medical education. Outdated information is widespread both in training programs and in the public sphere. Obtaining accurate information about FABMs is further complicated by the numerous period tracking or fertility apps available, because very few of these apps have evidence to support their effectiveness for identifying the fertile window, for achieving or preventing pregnancy.

**Conclusions:**

This article provides an overview of different types of FABMs with a published evidence base, apps and resources for learning and using FABMs, the role FABMs can play in medical evaluation and management, and the effectiveness of FABMs for family planning, both to achieve or to avoid pregnancy.

## Introduction

Women's interest in learning to track their menstrual or reproductive cycle has increased dramatically over the last couple of decades, both for health monitoring and family planning purposes (1–3). This interest has been paralleled and fueled by the development of over 500 mobile health applications intended for cycle tracking, more than triple the number from only 5 years ago (4–6). By working with trained instructors or via other educational programs, women can learn how to track their cycles and observe specific external signs or biomarkers that reflect normal and abnormal hormonal patterns and reproductive function (7, 8). Women or couples can also use this information for family planning purposes (9, 10). The purpose of this review is to provide an overview of current evidence about fertility awareness-based methods (FABMs) in the context of women's health, and for achieving or avoiding pregnancy.

Historically, FABMs were most commonly referred to as natural family planning (NFP), which is defined by the World Health Organization as “methods for planning for avoiding pregnancies by observation of the natural signs and symptoms of the fertile and infertile phases of the menstrual cycle” (11). Some couples combine their knowledge of the fertile and infertile phase with the use of other methods during the fertile phase, such as barrier methods or withdrawal (12). As discussed later in this paper, we refer to this as FABMs combined with other methods. The term fertility awareness-based methods highlights that these methods may be used for more than family planning purposes and in recent decades, there has been additional focus on the value of using this information for medical evaluation and treatment (7, 8, 13, 14).

The menstrual cycle is increasingly recognized as a vital sign of health that women should have the opportunity to learn to monitor beginning in adolescence (15). Just as with other vital signs pointing to disease states, recognition of variations in menstrual patterns can improve early identification of potential health concerns that could become more severe if a timely diagnosis and appropriate treatment are not made (7, 15, 16). With most FABMs, women track vaginal bleeding and patterns of cervical fluid secretions and/or other biomarkers of health or fertility, such as basal body temperature (BBT) or urinary hormone measurements. Most FABMs employ a paper or electronic chart, which serves as a daily diary of the woman's own observations. Paradoxically, however, only 4% of physicians have received any formal training in FABMs (17). In addition, only 6% of physicians have correct knowledge about the perfect and typical use effectiveness of FABMs to avoid pregnancy (18). Without formal training in reading the female reproductive cycle chart, physicians and other clinicians may miss important information about this vital sign of health when providing care to their patients.

This article discusses FABMs that are frequently used in North America (Table 1), resources for clinicians to learn about FABMs, the role of FABMs in understanding women's health, and the effectiveness of FABMs for achieving or preventing pregnancy. Our intent is to provide information that physicians and other clinicians can use to guide patients who may benefit from learning FABMs. We also aim to provide information to clinicians about how FABMs can help with diagnosis and treatment of women's health conditions, including common conditions underlying female subfertility.

**Table 1:** Overview of fertility awareness-based methods (FABMs).

**Method**	Biomarkers^*^	**Identifies estimated day of ovulation**	**Published evidence to achieve pregnancy**	**Published evidence to prevent pregnancy**	**Considerations**	**Teacher training**	**Web-based or mobile apps^†^**	**Other methods using similar approach to identify fertile window^††^**
Billings Ovulation Method^®^	CF	Yes	Yes	Yes^§^	Available through personal instruction^§^	Yes	NFP Charting; Ovulation Mentor; Fertility Pinpoint	Fertility Education and Medical Management (FEMM); Family of the Americas
Creighton Model FertilityCare System™	CF	Yes	Yes	Yes^§^	Available through personal instruction^§^	Yes	Fertility*Care* App	Justisse; NeoFertility
TwoDay^®^	CF	No	No	Yes	Not suitable for women with continuous vaginal discharge	Minimal	2 Day Method	
Sympto-thermal, Sensiplan^™^^||^	CF, BBT, CAL	Yes	Yes	Yes^§^	Available through apps & personal instruction^§^ Requires adaptation for anovulation (no change in temperature)	Variable	SymptoPro™; Kindara; Sympto; LilyPro; MyNFP LadyCycle; CyclePro Go	CCL-Couple to Couple League; SymptoPro™
Natural Cycles	BBT (uLH)	Yes	Yes	Yes	Requires ovulatory cycles (temperature)	NA	Natural Cycles app is US FDA approved as contraceptive^+^	
Marquette Method©	uLH, uE, CAL, (CF), (BBT)	Yes	Yes	Yes^§^	Available through online and personal instruction^§^ Requires use of Clearblue^®^ fertility monitor (cost)	Yes	Web-based charting^+^	
Dynamic Optimal Timing (DOT)™	CAL	No	No	Yes	Cycles need to be 20–40 days long	NA	CLUE Birth Control app is US FDA approved as contraceptive^+^	
Standard Days^®^	CAL	No	No	Yes	Cycles need to be 26–32 days long	Minimal	Cycle Beads	
Lactational Amenorrhea	Other	No	NA	Yes	Within first 6 months postpartum, no menstrual bleeding, totally breastfeeding	NA		

* *Biomarker abbreviations: CF, cervical fluid; BBT, basal body temperature; CAL, calendar calculations based on cycle length and/or prior days of ovulation; uLH, urine LH tests; uE, urinary estrogen metabolites. Parentheses indicate optional additional biomarker*.

† *Apps or web applications were included if they followed the same guidelines for identifying the fertile window as the FABM method they represent (5), but only those apps marked with ^+^ been directly evaluated for pregnancy prevention*.

†† *These similar methods have no peer-reviewed evidence for effectiveness for pregnancy prevention among their own users*.

§ *In pregnancy prevention effectiveness studies of the Billings, Creighton, Sympto-Thermal Method and in most studies of the Marquette method, couples learned the method from a trained instructor*.

|| *Sensiplan—sympto-thermal method with the strongest evidence base, but with limited availability in the US*.

## Physiology Underlying Fertility Awareness-Based Methods

**Figure 1:**
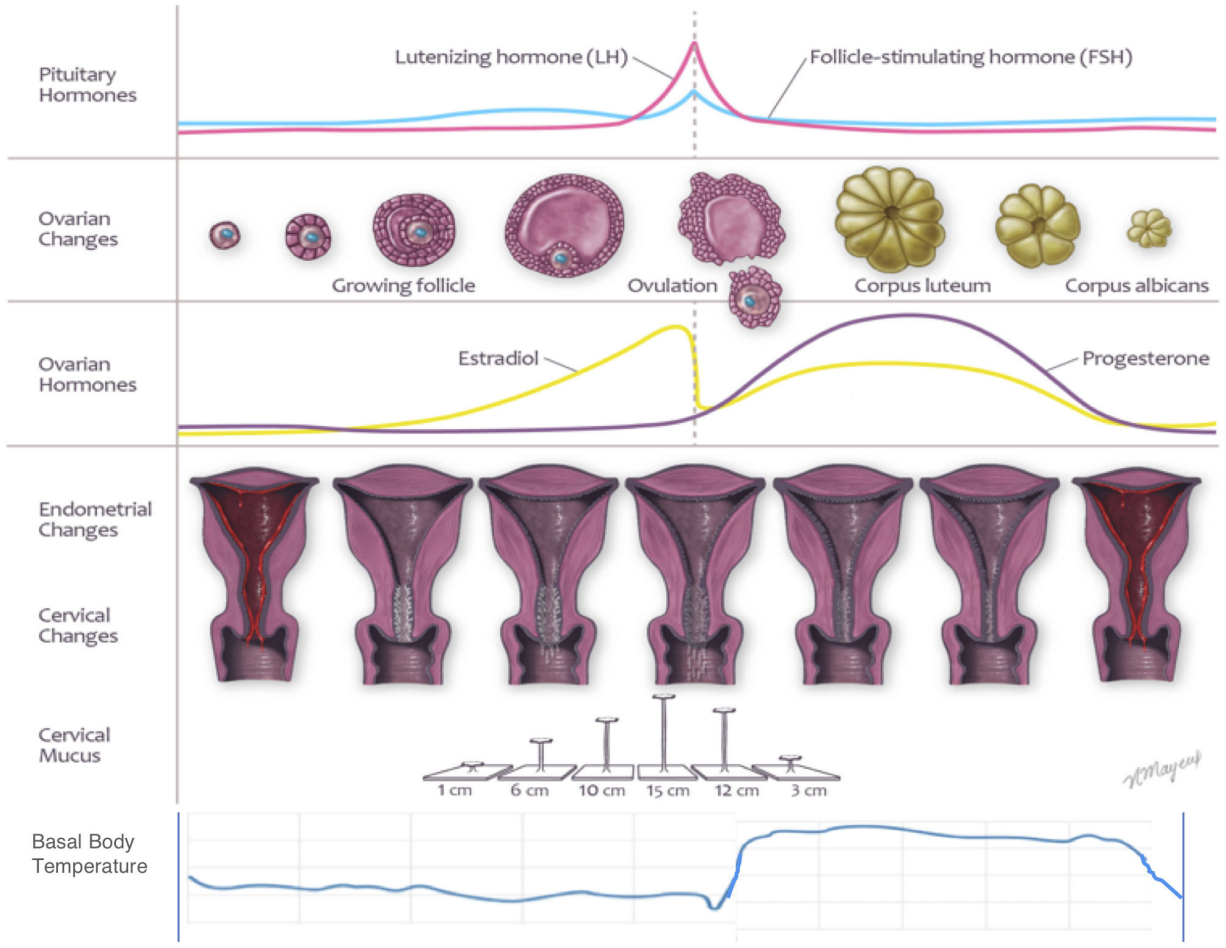
Physiologic changes of the female cycle.

FABMs arise from an understanding of how the normally functioning reproductive age female produces observable external biomarkers, or ovulation indicators, that reflect internal hormonal changes. Figure 1 illustrates the relationship between a female's reproductive organs, hormones, and cyclic changes in ovulation indicators, including cervical mucus or fluid secretions, luteinizing hormone (LH), and basal body temperature (BBT). The uterine cervix plays a key role in producing the different types of cervical fluid that perform important functions related to sperm storage, transport and fertilization (19–21). Changes in cervical fluid, LH and BBT are each useful to identify the occurrence and timing of ovulation, which is usually the central event of the menstrual cycle (22).

Although the onset of menses is used to identify the beginning of the cycle, the menstrual bleed, or “menstrual period,” actually marks the end of the previous ovulatory cycle. Then, under the influence of gonadotropin-releasing hormone, the pituitary secretes follicle stimulating hormone (FSH) in the follicular phase (7, 13). Rising levels and changes in pulse frequency of FSH stimulate the growth of ovarian follicles that produce estradiol and related hormones (7, 13). In addition to building up the endometrium, estradiol also acts on crypt cells in the cervix, which results in the production of fertile type E cervical mucus, which is clear, stretchy, and/or slippery in sensation (13, 22–24). When estradiol rises and reaches a threshold, mid-cycle, it triggers a luteinizing hormone (LH) surge that results in ovulation (7, 13, 25).

Ovulation only occurs on 1 day in each cycle and the ovum or ova will survive <12–24 h if not fertilized (7, 25). Type E cervical fluid produced under the influence of estradiol in the peri-ovulatory period is critical for the effective transport, nurturing and survival of sperm (22, 23, 26). The last day of fertile type E cervical fluid, designated the mucus peak day, is a good external marker, as ovulation occurs within 2–3 days of the mucus peak day 87–98% of the time (23, 26, 27). After ovulation, the luteal phase begins. The ruptured follicle transforms into the corpus luteum and begins to secrete progesterone and estradiol (7, 28). The secretion of progesterone causes the cervical fluid to become thick and impermeable (Type G or gestagenic cervical fluid), and results in a change in sensation, typically causing dryness (21–23, 29). Progesterone also increases the metabolic rate and leads to a rise in the basal body temperature (BBT) (7, 12). Finally, progesterone also converts the endometrium from proliferative to secretory to prepare for possible implantation. In the case of implantation of an embryo, human chorionic gonadotropin (hCG) is produced, which stimulates the ovary to continue producing progesterone and estradiol (30). If implantation does not occur, in the absence of hCG, the corpus luteum atrophies and progesterone levels drop, which results in the shedding of the endometrial lining (menstruation) and the next cycle begins (7, 25).

With regard to cycle lengths, the follicular or preovulatory phase is inherently more variable than the luteal or postovulatory phase (31, 32). When considering past cycle lengths and prior estimated ovulation dates, it is possible to use evidence-based calendar formulas to estimate the start and end of the fertile window; however, calendar formulas are not precise enough to provide reasonable estimates of the day of ovulation. It must be emphasized that most calendar formulas in popular use, and even in most apps, are oversimplified, not individualized, and are not evidence-based (5, 33).

Broadly, there are six different types or categories of FABMs, based on the biomarkers or fertility indicators that are used to identify ovulation and the fertile window (see Table 1 for overview of the types and the indicators used for each). These include cervical fluid (mucus) methods, BBT methods, urinary hormone methods, sympto-thermal methods, sympto-hormonal methods, and calendar-based (i.e., cycle length-based) methods. Finally, the lactational amenorrhea method is an effective natural method that a woman may use within the first 6 months post-partum as long as she has not had a return of menses and her baby is breastfeeding exclusively at the breast (34).

## FABMs for Women's Health

A woman's ovulatory function will vary normally throughout her reproductive years as part of healthy physiologic transitions, such as menarche, pregnancy, lactation, and menopause (35). Tracking of one or more indicators of ovulation can be used to detect and diagnose common underlying causes of ovulation disturbances. Menstrual cycle irregularities related to ovulation dysfunction are most commonly due to hormonal abnormalities, which may result from hypothalamic, pituitary, thyroid, adrenal, ovarian and metabolic disorders (7, 8, 15, 16). For example, hypothalamic disorders due to excessive exercise, disordered eating or stress may result in hypoestrogenic, anovulatory cycles and/or prolonged periods of amenorrhea (7, 13). Variations or changes in symptoms or parameters that women can observe through FABM charting are listed in Table 2, together with conditions that may underlie each of the patterns (26, 36–56). Two of these conditions, polycystic ovarian syndrome, and endometriosis, are each present in at least 10% of all women of reproductive age, and are among the most common underlying causes of subfertility (48, 57–59).

**Table 2:** Cycle parameters observable with fertility awareness-based methods and associated underlying health conditions.

**Symptom/observation**	**Possible underlying conditions**	**Selected references (first author, year, citation)**
Absent or infrequent ovulation; prolonged follicular phase; absent luteal phase	Hypothalamic amenorrhea; Polycystic ovarian syndrome; hyperprolactinemia; hypothyroidism	Saei Ghare Naz et al. (36) Barron (37) Gordon et al. (38)
Short luteal phase	Inadequate luteal function; stress; hyperprolactinemia; hyperandrogenemia; weight loss	Schliep et al. (39) Fatemi (40)
Low basal body temperature	Hypothyroidism	Hirata et al. (41)
Extended or continuous cervical fluid	Polycystic ovarian syndrome; high baseline estrogen; cervical ectropion	Shamim et al. (42) Najmabadi et al. (26) Baram et al. (43)
Limited quality or quantity of cervical fluid	Low follicular estrogen; endometriosis; prior cervical procedures	Stanford et al. (44) Hilgers (45) Dunson and Colombo (46)
Baseline elevated LH	Polycystic ovarian syndrome	Coyle and Campbell (47) Deswal et al. (48)
Premenstrual spotting	Endometriosis	Heitmann et al. (49)
Intermenstrual bleeding	Thyroid disease, hyperprolactinemia, Polycystic ovarian syndrome; endometrial polyp; endometritis; ovarian dysfunction	Koutras (50) Abdel Hamid et al. (51) Hickey et al. (52) Salim et al. (53) Smith et al. (54)
Dysmenorrhea	Endometriosis	Yeung et al. (55)
Postovulatory mood changes	Premenstrual dysphoric disorder, premenstrual syndrome	Hofmeister and Bodden (56)

## Medical Protocols Based on FABMs

There are several ways that FABMs can be used to enhance medical evaluation and treatment for women. (1) Women's observations on the FABM chart may suggest the presence of conditions that need further evaluation with diagnostic studies (Table 2). (2) Identifying the time of ovulation facilitates the scheduling and interpretation of time sensitive evaluations. For example, progesterone levels are very low prior to ovulation, and are normally at a maximum level 5–8 days following ovulation. Identifying ovulation allows the measurement of progesterone when it should be at its highest level (8). (3) Chart patterns may reflect intermediate outcomes from different types of fertility treatments. For example, a change from anovulatory to ovulatory cycles will be reflected in the woman's observation of her ovulation indicators (35).

Integrated medical evaluation and management protocols based on FABMs have been developed to address many women's health conditions that are related to the menstrual cycle, including subfertility or infertility. Natural Procreative Technology (also known as NaProTechnology) is a set of evaluation and treatment protocols developed based on women charting with the Creighton Model FertilityCare System (8, 45). It includes medical and surgical components. The Reproductive Health Research Institute (RHRI) has also published a set of medical evaluation and treatment protocols for women's health conditions, which are often related to FEMM (Fertility Education and Medical Management), but can also be used with any FABM that identifies ovulation accurately (7, 35, 60). A detailed or critical review of the components of each of these protocols is beyond the scope of this article; however, several resources for continuing medical education in FABMs are now available (see Table 3).

**Table 3:** Continuing medical education (CME) resources for medical applications of fertility awareness-based methods.

**Organization**	**Introductory courses**	**Professional meetings**	**FABMs emphasized**	**Website**
American Academy of FertilityCare Professionals (AAFCP)	No	Yes	Creighton Model Fertility*Care* System and NaProTechnology	aafcp.net
Billings Ovulation Method Association-USA (BOMA)	Yes	Yes	Billings Ovulation Method	www.boma-usa.org/health-professionals.html
Fertility Appreciation Collaborative to Teach the Science (FACTS)	Yes	Yes	All	www.FACTSaboutFertility.org
Reproductive Health Research Institute (RHRI)^*^	Yes	No	Fertility Education and Medical Management (FEMM), Billings Ovulation Method	femmhealth.org/professional-education/medical-training/
International Institute for Restorative Reproductive Medicine (IIRRM)	No	Yes	All	iirrm.org; iirrma.org
NeoFertility	Yes	No	NeoFertility	www.chartneo.com
St. Paul VI Institute for the Study of Human Reproduction	Yes	No	Creighton Model Fertility*Care* System and NaProTechnology	popepaulvi.com

* *RHRI medical protocols may be used in conjunction with any FABM that tracks ovulation (e.g., Billings, Creighton, FEMM, STM)*.

## FABMs for Achieving Pregnancy

FABMs enable couples to achieve pregnancy by identifying the relatively few days during the female cycle when sexual intercourse will likely result in fertilization. This window of time, defined as the fertile window, usually begins about 5 days prior to ovulation, and ends within 12–24 h after ovulation (22, 61, 62). When the fertile window is estimated by cervical fluid and other biomarkers, it is usually varies from 1 day (in some subfertile populations) to more than 6 days long (26, 44, 61–65). In couples without subfertility, the highest probability of pregnancy per cycle is ~20–40%, depending on the characteristics of the population, including age and parity. It occurs when couples have intercourse 1–2 days *before* ovulation, particularly on days with the greatest estrogenic qualities of cervical fluid (clear, stretchy, slippery fluid), which optimizes sperm survival and transport (28, 64, 66). Data are sparse and mixed as to whether frequent intercourse decreases or actually increases overall sperm motility and concentration, and how this may impact the probability of pregnancy (67–69).

When couples regularly engage in acts of intercourse without attention to timing, approximately 85% of them will conceive by the end of 1 year (62). When couples can identify their fertile window and engage in fertility-focused intercourse, evidence suggests they can achieve similar pregnancy rates in less time. A number of studies have examined fertility focused intercourse with several different FABMs (Table 4) (70–79). Overall, these studies suggest that 85–90% of couples without subfertility can conceive within 6 months through fertility focused intercourse. Public awareness of the benefits of using FABMs to conceive is becoming more widespread in a recent large online study of couples in the USA and Canada trying to conceive, 75% of women were already using one or more FABM indicators to try to conceive (albeit not necessarily accurately), and 73% were using a menstrual and/or fertility tracker app (71).

**Table 4:** Fertility awareness-based methods and time to pregnancy in women or couples with no known subfertility trying to conceive.

***N***	**Population**	**Mean women's age (years)**	**Study design**	**Follow-up duration**	**FABM**	**Major findings**	**References**
5,376	Sweden, UK, USA	31.8	Cohort	Up to 12 cycles	Natural Cycles	Cumulative pregnancy at 12 cycles 74%; median 4 cycles to pregnancy; for women <35 years with regular cycles, 95% and median 2 cycles to pregnancy	Favaro et al. (70)
8,363	North America, recruited online	29.9	Cohort	Up to 1 year	Multiple	Among women using an app together with cervical fluid, basal body temperature, and/or urine LH: fecundability ratio^*^ 1.21–1.26	Stanford et al. (71)
785	UK, recruited online	30	Randomized trial	Up to 2 cycles	Clearblue Connected Ovulation Test System^®^	After 2 cycles, 36.2% pregnant in FABM arm; 28.6% pregnant in control arm	Johnson et al. (72)
2,874	84% from Sweden; online	28.1	Cohort	Up to 13 cycles	Natural Cycles	Recent use of hormonal contraception: mean 3.7 cycles to pregnancy; no recent hormonal contraception: mean 2.3 cycles to pregnancy	Berglund Scherwitzl et al. (73)
256	North America	29.2	Cohort	Up to 24 cycles	Marquette	Cumulative pregnancy rates by cycle 12 was 83% (monitor only), 72% (mucus only) and 75% (monitor and mucus)	Bouchard et al. (74)
124	Women recruited online	29.5	Cohort	Up to 1 year	Marquette	With intercourse on high fertile days, 87% pregnant at 1 year	Mu and Fehring (75)
69 in CrM group	Women in Utah, all parous	28.2	Randomized trial	Up to 9 months	Creighton Model	Cumulative pregnancy 93% by cycle 7 of trying to conceive in CrM group; no significant difference from non-CrM group; randomized trial was confounded by instruction to avoid pregnancy in first cycle	Stanford et al. (76)
331	Women in North Carolina	30% >35 years	Cohort	Up to 12 months	Cervical fluid	Among women consistently observing cervical fluid: fecundability ratio^*^ 2.3	Evans-Hoeker et al. (77)
346	German couples	29.0	Cohort	Up to 6 months	Sensiplan	Cumulative pregnancy proportion by 6 months: 92%	Gnoth et al. (78)

* *The fecundability ratio is the relative probability of conceiving per menstrual cycle; a higher fecundability ratio results in shorter time to pregnancy*.

## FABMs for Subfertility

In women with cycle abnormalities or couples with subfertility or infertility, the female cycle chart may help a couple to identify a less frequent or narrower fertile window when trying to conceive (43, 79). It may also serve as a tool for clinicians trained in restorative reproductive medicine (RRM) to guide the work-up and management of multiple underlying causes of subfertility or recurrent pregnancy loss (7, 8, 50–53, 80).

There are several studies that document the outcomes of using FABMs to address subfertility, either with or without medical intervention (Table 5) (80–85). Four of these studies arise from practices of family physicians utilizing RRM techniques in addition to the FABM charting by the women and couples (80–83). Currently randomized comparisons of RRM vs. conventional fertility treatments are not available. It should also be noted that there are few randomized trials that compare different types of fertility treatments to each other. Most randomized trials involve adjustments within a particular treatment (e.g., different protocols for *in vitro* fertilization), rather than comparisons between different types or classes of treatment (86, 87). There are also practical and ethical considerations in studies of fertility therapies or family planning methods, in that women or couples who wish to choose which therapy or method of family planning they use may not be willing to be randomized to a different method.

**Table 5:** Effectiveness of fertility awareness-based methods in women or couples with subfertility trying to conceive.

***N***	**Population**	**Mean women's age (years); % prior birth; prior time trying**	**Study design**	**FABM**	**Medical interventions**	**Live birth or pregnancy rates**	**References**
370	Massachusetts, USA	34.8 years 27% 2.7 years	Cohort	Creighton Model or Sympto-thermal	Multiple medical interventions; subset had surgery for endometriosis	Adjusted 29% live birth rate with up to 2 years of treatment; 40% for women with BMI <25	Stanford et al. (81)
384	Australia	33.1 years Unknown prior birth 51% had tried >1 year	Cohort	Billings	None stated	Women with good cervical fluid: 76% pregnant in 2 years; poor cervical fluid: 44% in 2 years	Marshell et al. (82)
403	Ireland	37.2 years 22% 5.8 years	Cohort	Creighton Model	Multiple medical interventions; subset had surgery for endometriosis	Adjusted 32% live birth rate with up to 2 years of treatment; 33% for women with 3 or more ART attempts	Boyle et al. (80)
187	Heidelberg, Germany	34.7 years 15% 3.5 years	Cohort	Sensiplan	None	Adjusted pregnancy rate 38% at 8 months	Frank-Herrmann et al. (83)
108	Toronto, Canada	35.4 years 20% 3.2 years	Cohort	Creighton Model	Multiple medical interventions; subset had surgery for endometriosis	Adjusted 66% live birth rate with up to 2 years of treatment	Tham et al. (84)
1,072	Galway, Ireland	35.8 years 24% 5.6 years	Cohort	Creighton Model	Multiple medical interventions; subset had surgery for endometriosis	Adjusted 53% live birth rate with up to 2 years of treatment	Stanford et al. (85)

## FABMs for Preventing Pregnancy

With identification of the fertile window, couples can modify their sexual behavior to avoid pregnancy, e.g., by abstaining from sexual contact during the fertile window. Two recent systematic reviews have summarized the evidence for pregnancy rates when FABMs are used to avoid pregnancy, based on cohort studies from around the world (9, 10). From these reviews, we present a summary overview of the studies and their pregnancy rates that were judged of reasonable methodologic quality in at least one of the reviews, were published since 1990, had at least 100 women, and which involve FABMs that are currently readily available in North America (see Table 6). With regard to the Marquette Model, we only included those study populations using the Clearblue^®^ monitor. We also included three more recent studies that we believe may meet the same level of methodologic quality, but which have not yet been vetted in a systematic review. Correct use pregnancy rates (also called “perfect use” pregnancy rates) are observed during consistent and correct application of the FABM to avoid pregnancy (101) (These rates have often been calculated incorrectly based on all participant time in the study; we present in the table those which have been calculated correctly based on the participant time of correct use). Typical use pregnancy rates are observed during actual use of the FABM to avoid pregnancy, including both correct and incorrect use. Because the use of FABM to avoid pregnancy requires adaptations in sexual behavior, and because strength of motivation to avoid pregnancy may vary between populations or change over time, there is a difference between pregnancy rates with correct use and typical use, which may vary substantially based on underlying characteristics and motivations of the population being studied (102). Furthermore, some studies assessed pregnancy intentions only once at the beginning of the study, whereas others did so at the beginning of each cycle.

**Table 6:** Pregnancy rates for fertility awareness-based methods used to avoid pregnancy^*^.

***N***	**Method**	**Correct use pregnancy rate^†^ (95% C.I.)**	**Typical use pregnancy rate^†^ (95% C.I.)**	**Study participant characteristics**					**References**
				**Woman's age**	**Study location**	**Woman's education**	**Relationship status**	**Previous pregnancy**	
900	Sympto-Thermal Method (Sensiplan)™	0.4 (0.1–1.6)	1.8 (1.0–2.6)	<30 years 63%	Germany	Secondary 64% University 25%	Married 36% Unmarried 63%	Prior birth 48%	Frank-Hermann et al. (88)
197	Marquette Method© (Clearblue^®^ only)	0	6.8	Mean 29.7 years	USA	NR	NR	Mean n children 1.8, 2.1	Fehring et al. (89)
212		NR	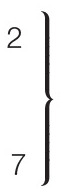	Mean 30.3 years	USA	College graduates 80%	NR	Mean n children 2.4	Fehring et al. (90)
333	Marquette Method© (Clearblue^®^ and cervical fluid)	NR							
195		2.1	14.2	Mean 29.5 years	USA: Atlanta, Madison, Milwaukee, St. Louis;	At least high school 90%	NR	NR	Fehring et al. (91)
2,059	Billings Ovulation Method^®^	1.1 (0.5–1.7)	10.5 (9.1–11.9)	Mean 26.2 years; Range 15–35	Rural India	Illiterate 32% Up to 8th−24% High level 19%	Married 100%	Mean # of pregnancies 2.5	Bhargava et al. (92)
869		3.4	22.8	Mean 29.2 years	New Zealand, India, Ireland, Philippines, El Salvador	Illiterate 13% University graduate 9%	Married or cohabiting 100%	Prior birth 100%	Trussell and Grummer-Strawn (93)
701	Creighton Model FertilityCare™ System	NR	17.1^††^	20–34 years 88%	Houston, USA	College graduate 58%	Married or engaged 93%	NR	Howard and Stanford (94)
450	Two Day Method^®^	3.5 (1.4–5.5)	13.7 (9.9–17.3)	30 years or older 48%	Guatemala, Peru, Philippines	Minimal 27% Technical/ university 33.4%	NA	Prior birth 100%	Arevalo et al. (95)
478	Standard Days Method^®^	4.8 (2.3–7.1)	12.0 (8.5–15.3)	Mean 29.4 years	Peru, Bolivia, Philippines	At least some secondary education 85%	Married or cohabiting 100%	Prior birth 99%	Arevalo et al. (96)
301		NR	11.2	<30 years: 59%	Guatemala	Illiterate 32% >6 years education 16%	Married or in union for at least 1 year	Prior birth 100%	Burkhart et al. (97)
12,247	Natural Cycles^§^	2.0 (1.3–2.8)	7.1 (6.5–7.7)	Mean 30 years	UK	University Degree 83%	In a relationship 83%	Prior birth 16%	Pearson et al. (98)
5,879		2.0 (0.9–3.0)	7.2 (6.4–8.1)	Mean 30 years	USA	University Degree 84%	In a relationship 86%	Prior birth 17%	Pearson et al. (99)
718	Dynamic Optimal Timing^™^^§^	1.0 (0.9–2.9)	5.0 (3.4–6.6)	Mean 29 years	USA	NR	Married 27%	Prior pregnancy 52%	Jennings et al. (100)

*NR, not reported*.

* *Includes studies published since 1990 with over 100 participants that were rated in a systematic review as Level 1 (9) or had 7 or more high quality criteria (of 13 possible) (10). Marquette Method studies include only the study populations that used the Clearblue^®^ Monitor*.

† *Pregnancy rates are per 100 women or couples for 1 year of use. Some are calculated by life table, and some by Pearl Rate*.

†† *Total pregnancy rate, which includes some intended pregnancies*.

§ */sup>These studies were published after the systematic reviews, and have not undergone a standardized review for quality*.

With correct use to avoid pregnancy, the pregnancy rate is <5 per 100 women years for all methods included, and for some methods, it is <1% (Table 6). Typical use pregnancy rates vary depending on the characteristics of the population studied, and at least to some extent the individual method, ranging from about 2 to 23 per 100 woman-years, with the majority of studies showing typical use pregnancy rates of <15 per 100 woman-years.

It's important to recognize that pregnancy rates reported in studies to avoid pregnancy may not necessarily correlate with real world pregnancy rates, as effectiveness depends on adequate training or instruction, user motivation for correct use to avoid pregnancy, and sufficient cooperation or support from the male partner (10, 12, 103). To date, most FABM effectiveness studies for pregnancy prevention have been done with couples who learned the method from a trained instructor, with the exception of the Natural Cycles and DOT apps, which have electronic instruction resources (98, 99). It should also be noted that while the actual fertile window is generally considered to be about 6 days in healthy couples, there is some normal variability in the biomarkers, which means that the observed fertile window for FABMs to avoid pregnancy is usually about 12 days that are considered potentially fertile (26, 31, 64, 104).

It is more difficult to identify the beginning of the fertile window than the end of it, in part because of the inherent variability of the length of the follicular phase. Therefore, for couples who wish to have the least possible chance of pregnancy while using an FABM, it is prudent to recommend that they restrict intercourse to the postovulatory infertile phase, and that they consider using two indicators to confirm the end of the fertile window (10, 91, 105, 106). For example, the sympto-thermal method uses the mucus peak and the BBT shift combined to confirm the end of the fertile window (12, 88). As another example, the mucus peak and postovulatory measurement of progesterone may also confirm the end of the fertile window (10, 106, 107). This may result in a longer period of time when couples consider themselves to be potentially fertile.

## FABMs Combined With Other Methods

The concomitant use of barrier methods (e.g., condoms) or withdrawal during fertile days may influence pregnancy rates, in comparison to abstinence from genital contact during the fertile window. There are a few studies that have examined this question systematically. In a study of the Standard Days Method (*n* = 373), the correct use pregnancy rate with abstinence in the fertile time was 4.8% at 1 year, while the correct use pregnancy rate including barriers or withdrawal during the fertile time was 5.7% (96). Similarly, in a study of the TwoDay Method (*n* = 450), the correct use pregnancy rate with abstinence in the fertile time was 3.5%, while the correct use pregnancy rate including barriers or withdrawal during the fertile time was 6.3% (95). In a study of 900 women using Sensiplan, the 13-cycle cumulative typical use pregnancy rates were 1.6% for Sympto-Thermal only, and 2.0% for occasional use of barriers in the fertile time (88). Overall, these data do support the logic that use of a barrier method or withdrawal during a fertile day should be expected to have at least a slightly higher chance of pregnancy than no sexual contact during that same fertile day.

## Cycle Tracking Apps

In the last decade there has been an explosion in the number of fertility apps for smart phones and other mobile devices, available for women to track their cycle, with more than 500 apps available via Google and the Apple app store when using the keyword *fertility* to search for apps (6). Although many apps claim to be useful for avoiding or achieving pregnancy, a 2016 systematic review of apps marketed for avoiding pregnancy demonstrated that the large majority are not concordant with evidenced-based methods of fertility awareness. The few apps that were rated highly were associated with established FABM methods (see Table 1) (5). Similar results were reported in a 2020 scoping review, namely that few apps accurately predict the fertile window (108). To date, two apps, Natural Cycles and Clue, the former based primarily on basal body temperature, and the latter using the dynamic optimal timing (DOT) algorithm, have received FDA clearance for use as a contraceptive device (99, 100). Both apps support an approach of FABM combined with barrier methods, as they stipulate that correct use includes the possible use of barrier methods on fertile days. One app interprets urine estrogen metabolites and LH to define the fertile window, and has been shown effective for trying to conceive (see Table 4) (72). Additional apps are available or being developed that may integrate artificial intelligence to interpret hormones or metabolites in urine, including estradiol, LH, and progesterone, but as yet there is no published research on their effectiveness to avoid pregnancy or to conceive (109).

## Relationship Influences

FABMs or natural methods are unique among family planning options, in the level of encouraging understanding, involvement or assent from both partners, and communication between them (9, 12, 103, 110). As behavioral methods of family planning, FABMs rely on people learning to track the observable female biomarkers on a daily basis to determine whether they may be fertile and when they are not (9, 12). They can then share this information with their partner and depending on their family planning goals follow the rules of their chosen method for preventing or achieving pregnancy (111). These methods may positively influence relationships and body literacy. One study of over 2500 sympto-thermal users found large majorities of women and men felt NFP improved their relationship and sex-life, and three-fourths of them were satisfied with how often they had sexual intercourse. Fully 95% of women reported using a natural method improved their body literacy (103).

## Learning FABMs

To maximize effectiveness of any FABM, it is important that people receive adequate instruction, which clearly identifies biomarkers of interest and how they may be tracked to understand fertility. We believe this is particularly true for using FABMs in medical applications. To date, most studies of FABMs to avoid pregnancy or to conceive have delivered this instruction via trained teachers, usually in person (9). More recently, online models of instruction have proven effective for some methods (102). Some simpler methods have delivered their instruction through online resources, such as videos (11, 99, 100).

## Conclusion

FABMs serve as a useful tool for people to track daily external observations that reflect ovulation and the internal hormonal changes women experience throughout their cycle. Physicians and other clinicians may learn to interpret the female cycle chart to identify potential abnormalities of the menstrual cycle and inform a differential diagnosis and management plan to address a range of reproductive health issues, such as abnormal uterine bleeding, subfertility, and other conditions associated with abnormalities in ovulation or reproductive hormone levels. When clinicians are knowledgeable about the range of FABMs and their effectiveness, they can also offer patients a wider array of options for seeking pregnancy or avoiding pregnancy (both aspects of family planning), which will meet the needs of more people. Adding FABMs to the mix of available contraceptive methods has been demonstrated to expand the proportion of women using family planning, without any increase of unplanned pregnancy rates (112). Unfortunately, most physicians are currently not well-versed in modern FABMs, the science underlying their use, or the medical applications of these methods (18). This article offers an introduction to FABMs and their medical applications for physicians and other clinicians. For more information, we encourage our colleagues to pursue continuing medical education options in restorative reproductive medicine, such as those outlined in Table 3.

## Author Contributions

MD and JS designed the review, conducted the literature search, and drafted the initial manuscript. CP provided a critical review of the manuscript and assisted with the creation of the tables. PV assisted with writing the manuscript and provided critical review of the content. All authors reviewed and approved the final manuscript.

## Funding

This project is supported by the Health Resources and Services Administration (HRSA) of the U.S. Department of Health and Human Services (HHS) as part of an award totaling $ $1,572,177 over 5 years with zero percentage financed with non-governmental sources. The contents are those of the author(s) and do not necessarily represent the official views of, nor an endorsement, by HRSA, HHS or the U.S. Government.

## Author Disclaimer

The contents are those of the author(s) and do not necessarily represent the official views of, nor an endorsement, by HRSA, HHS or the U.S. Government.

## Conflict of Interest

MD serves as a paid part-time Director of the Fertility Appreciation Collaborative to Teach the Science (FACTS), a collaborative project of the Family Medicine Education Consortium. JS serves without compensation on the Boards of the International Institute of Restorative and Reproductive Medicine and the Fertility Care Centers of America. CP serves on the board of the International Board of Lactation Consultant Examiners. PV serves as the medical director of the Reproductive Health Research Institute.

## Publisher's Note

All claims expressed in this article are solely those of the authors and do not necessarily represent those of their affiliated organizations, or those of the publisher, the editors and the reviewers. Any product that may be evaluated in this article, or claim that may be made by its manufacturer, is not guaranteed or endorsed by the publisher.
